# An update on the cerebellar subtype of multiple system atrophy

**DOI:** 10.1186/s40673-014-0014-7

**Published:** 2014-10-10

**Authors:** Ludovico Ciolli, Florian Krismer, Ferdinando Nicoletti, Gregor K Wenning

**Affiliations:** Sapienza University, Via di Grottarossa, 1035-00189 Rome, Italy; Department of Neurology, Innsbruck Medical University, Anichstrasse 35, A-6020 Innsbruck, Austria; IRCSS NEUROMED, Pozzilli, Isernia Italy; Department of Physiology and Pharmacology “Vittorio Erspamer”, Sapienza University, School of Medicine and Psychology, Rome, Italy

**Keywords:** Multiple system atrophy, Cerebellar type, Idiopathic late onset cerebellar ataxia, Sporadic adult onset ataxia

## Abstract

Multiple system atrophy is a rare and fatal neurodegenerative disorder characterized by progressive autonomic failure, ataxia and parkinsonism in any combination. The clinical manifestations reflect central autonomic and striatonigral degeneration as well as olivopontocerebellar atrophy. Glial cytoplasmic inclusions, composed of α-synuclein and other proteins are considered the cellular hallmark lesion. The cerebellar variant of MSA (MSA-C) denotes a distinctive motor subtype characterized by progressive adult onset sporadic gait ataxia, scanning dysarthria, limb ataxia and cerebellar oculomotor dysfunction. In addition, there is autonomic failure and variable degrees of parkinsonism. A range of other disorders may present with MSA-C like features and therefore the differential diagnosis of MSA-C is not always straightforward. Here we review key aspects of MSA-C including pathology, pathogenesis, diagnosis, clinical features and treatment, paying special attention to differential diagnosis in late onset sporadic cerebellar ataxias.

## Introduction

Multiple system atrophy (MSA) is a rare, sporadic, progressive, neurodegenerative disorder combining features of parkinsonism, autonomic dysfunction and cerebellar and pyramidal signs. MSA can be further classified in parkinsonian-type MSA (MSA-P) and cerebellar-type MSA (MSA-C) according to the predominant motor symptoms at evaluation.

MSA has a prevalence of 1.9-4.9 cases per 100000 [1,2] and an incidence of 0.6/100000, raising up to 3/100000 in people older than 50 years [3].

MSA-P is the more common variant in Europe and in USA, accounting for about 65% of all cases [4-6]. In the Japanese population MSA-C is present in 83.8% of MSA patients at first examination and in 48.6% of patients at last follow-up [7]. This difference could be caused by, not yet fully understood, genetic predisposition and environmental influences in the pathogenesis of the disease. Median survival ranges from 6 years to about 9 years [6,8,9].

Here, we aim to provide an update on the pathology, pathogenesis, diagnosis, clinical presentation and latest therapeutic development in MSA-C. We searched the following terms on PubMed: *“multiple system atrophy”, “idiopathic late onset cerebellar ataxia”, “sporadic adult onset ataxia”*. In addition, reference lists in review papers were systematically checked for relevant references. Only papers in English were reviewed.

## Review

### Pathology

Twenty years ago Papp and coworkers identified, for the first time, the argyrophilic filamentous aggregates localized in the cytoplasm of oligodendrocytes that were common to all MSA variants. These inclusion bodies were subsequently termed glial cytoplasmic inclusions (GCIs) or Papp-Lantos bodies [10]. GCIs are typically associated with gliosis and neuronal loss in the basal ganglia, cerebellum, pons, inferior olivary nucleus, and spinal cord. Frequently a striatonigral (SND) or olivopontocerebellar (OPCA) pattern of atrophy can be defined. Patients can present either a balanced damage in both regions or a predominant involvement of one system over the other. In both cases, the pathological alteration determines the clinical phenotype, i.e. clinically diagnosed MSA-C often reflects underlying OPCA [11-14]. In MSA pathological alterations are not limited to OPCA and SND but many other brain regions can be involved. Degeneration of several autonomic nuclei in the brainstem and spinal cord is traditionally believed to account for autonomic failure in MSA [15-17]. However, a growing body of evidence suggests that post-ganglionic denervation also occurs in MSA patients, and may be involved in the pathogenesis of dysautonomia [18-21]. Accordingly, the sudomotor nerve density in the sweat glands has been found to be reduced in patients with MSA as compared to healthy controls. Interestingly, peripheral nerve degeneration has been found in the early course of the disease, and may, therefore, be independent of degeneration of autonomic CNS nuclei [21].

In the late 1990’s, α-synuclein was identified as the main component of GCIs [22-25]. Widespread GCIs cannot be found in other diseases and are always present in patients with MSA, regardless of the clinical phenotype. Their assessment is considered as the only reliable criteria for the diagnosis of definitive MSA [26,27]. Additionally, neuronal cytoplasmic inclusions (NCIs) and neuronal nuclear inclusions (NNIs) can be found in MSA as well, however, they are only of limited diagnostic value [28].

Of note, however, α-synuclein is not the only component of GCIs. Ubiquitin, tau, p25α, members of the heat shock protein family, dopamine and c-AMP-regulated phosphoprotein-32 (DARPP-32), and many other proteins have been detected in different proportion in GCIs [17]. Of interest, p25α, a normal constituent of myelin sheets in healthy neurons, seems to have a facilitatory effect on GCIs formation, and its dislocation from the axons to the soma of oligodendrocytes [29] might therefore have a causative role in α-synuclein aggregation [30].

### Pathogenesis

Aberrant protein aggregation and dislocation can enhance neuronal demise [31,32] by disrupting the cytoskeleton [33]. While NNIs and NCIs directly damage neurons, GCIs primarily promote oligodendrocytes death [34-36], thereby causing secondary neurodegeneration. This assumption is also supported by evidence that GCI density correlates with disease duration and neuronal loss [13].

The most accredited theory linking oligodendrocyte damage with neuronal death focuses on disruption of the crosstalk between these cells. Oligodendroglial dysfunction results into an abnormal synthesis and release of trophic factors and other signal molecules, thereby triggering neuronal apoptosis [13,37].

**Figure 1:**
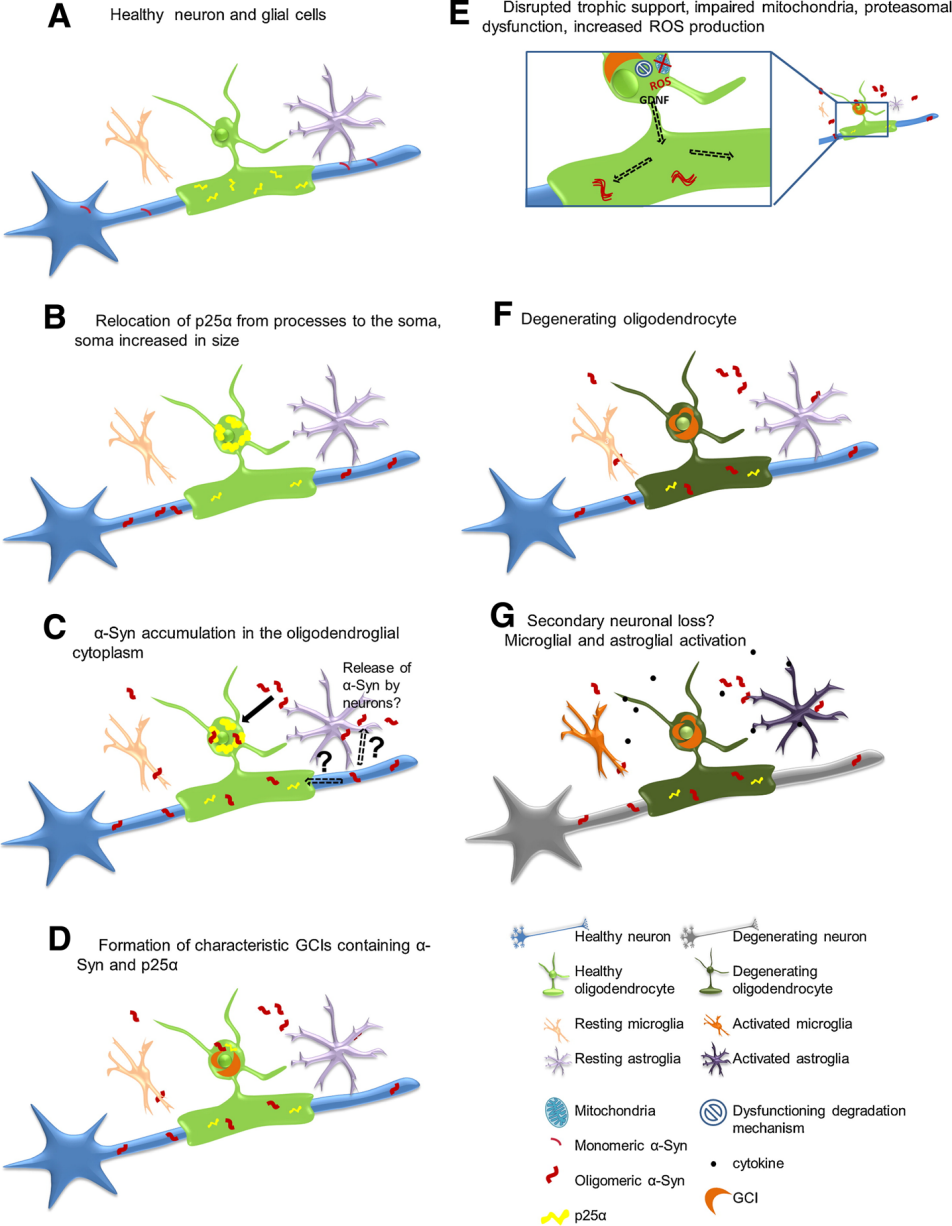
**Possible pathological a-Syn-spreading and accumulation mechanism leading to neurodegeneration. (A)** Healthy neuron, oligodendrocyte, microglia and astrocyte, p25a mainly located in the myelinating oligodendroglial processes, monomeric a-Syn present in presynaptic nerve terminals. **(B)** Relocalisation of p25a from the processes to the soma, inclusion formation and swelling of the oligodendroglial soma. **(C)** Oligomeric a-Syn accumulation in the oligodendroglial cytoplasm, the exact source of a-Syn remains to be investigated. Possible hypotheses include exocytosed a-Syn from neurons and uptake into oligodendrocytes by cell-to-cell propagation or upregulation of a- Syn expression in oligodendrocytes themselves. In addition, axonal a-Syn may be taken up by the dysfunctional oligodendroglial myelin compartment. **(D)** a-Syn aggregates form insoluble half-moon shaped GCIs characteristic for the disease. **(E)** Disruption of trophic support (e.g. GDNF), mitochondrial failure, increased production of reactive oxygen species (ROS) and proteasomal dysfunction occur. **(F)** Oligodendrocytes suffer from severe distress and will eventually degrade. **(G)** Activation of micro/astroglial cells by cytokines released from the damaged oligodendrocytes, proposed secondary neuronal loss potentially due to lack of trophic support, ROS production, proteasomal failure and pro-inflammatory environment. Reproduced with the courtesy of Elsevier.

Dysfunction of the mitochondrial respiratory chain may also contribute to the pathophysiology of MSA, as suggested by the evidence that variants in the *COQ2* gene that reduce the function of parahydroxybenxzoate-polyprenyltransferase (an enzyme necessary for the biosynthesis of coenzyme Q_10_) are associated with an increased risk of developing MSA [38]. Mitochondrial dysfunction leads to an excessive production of reactive oxygen species (ROS) [39,40], which have been implicated in the pathogenesis of MSA-associated neuronal damage [41,42]. ROS species are also generated by activated microglia, together with other damaging factors, such as nitrogen species and cytokines [43-45] (Figure 1) [17]. Microglial activation can be partially explained by an aberrant expression of Toll-like receptors (TLRs) in brain regions involved in OPCA and SND [46]. An imbalance of TLR signaling could enhance MSA-related brain injury by promoting pro-inflammatory signals [47,48].

In spite of the recent advances in the understanding of the pathogenesis of MSA, many questions remain unanswered. The influence of environmental and genetic factors remain unresolved in the western hemisphere. Although early reports suggested an association between MSA and agricultural work, the role of toxic substances could not be unequivocally proven [33]. Genetic inheritance is uncommon and there are few reports of familiar MSA in the literature [49-51]; however, the genetic background is important and single nucleotide polymorphisms (SNPs) of the genes encoding α-synuclein [52-54], the prion protein [55], and loss of function mutations of the phenylbenzoate-polyprenyltransferase were shown to be associated with an increased risk of MSA [38].

The origin of the α-synuclein found in GCIs is still unknown. Miller and coworkers failed to detect the expression of the gene coding for α-synuclein (the *SNCA* gene) in oligodendrocytes using double-labeling *in situ* hybridization technique [56]. Recently, however, α-synuclein mRNA could be measured in oligodendrocytes by laser-capture microdissection technique. mRNA levels in oligodendrocytes did not differ between MSA patients and healthy controls, although there was a trend to an increase in MSA patients [57]. Proteosomal and autophagic degradation of α-synuclein has also been found to be impaired in affected oligodendrocytes [17,56,58].

An increased uptake of α-synuclein from the extracellular environment, particularly from the cerebrospinal fluid (CSF) [59], may also contribute to GCI formation and propagation [60,61]. Reyes et al. (2014) [61] have shown that oligodendrocytes grafted in the striatum of rats overexpressing human α-synuclein, take-up the protein from surrounding axon terminals. This evidence suggests a prion-like propagation of α-synuclein in MSA, similarly to what hypothesized for Parkinson’s disease [62,63].

### Clinical features

MSA-C patients develop the first motor or autonomic symptoms at the mean age of 56 years [64,65]. Postmortem confirmed MSA-C has never been reported in patients younger than 30 years. If symptoms appear for the first time in patients older than 75 years, the diagnosis of MSA should be questioned [27]. Motor symptoms in MSA-C are often preceded by autonomic and other non-motor features [15,64]. Erectile dysfunction and urinary problems, such as incontinence and urinary retention, are frequently the first manifestation of autonomic failure in MSA-C [15,64,66,67]. Postural faintness tends to occur later [15,64,66,68]. Other symptoms of autonomic failure in MSA-C include reduced sweating and constipation. Recently, Iodice and colleagues (2012) [69] reported that the most common cerebellar feature at motor presentation is ataxic gait, followed by dysarthria, limb ataxia and gazed-evoked nystagmus. Although nystagmus is uncommon, other oculomotor abnormalities, such as jerky pursuit, square wave jerks, and dysmetric saccades are frequently observed in the early course of the disease [27]. Hyperreflexia and positive Babinski sign are classical manifestations of pyramidal tract degeneration and are frequently assessed in MSA-C patients.

Parkinsonism occurs about 5 years after the onset of MSA-C [70] and is usually characterized by an akinetic-rigid syndrome. Parkinsonian symptoms in MSA-C patients may respond to levodopa (L-DOPA) in up to 51% of cases, however, the effect is often transient and some patients also develop L-DOPA-induced dyskinesias [65].

Craniocervical dystonia is a common cause of postural aberrations such as camptocormia and Pisa syndrome. Dystonic involvement of face, hands and feet is also possible. Laryngeal abductor palsy resulting in stridor, can also be present. It commonly appears late in the disease course and is a possible cause of sudden death [71].

Ataxia, parkinsonism and postural impairment all contribute to gait instability, occurring early after the disease onset [64,72].

Microcirculatory abnormalities are common in MSA-C patients and are responsible for the so called “cold hand sign” [73-75].

Tison and colleagues [76] reported that about 30% of patients with MSA-C experience pain. The most common form is musculoskeletal pain, followed by sensory and dystonic pain.

Almost all patients with MSA develop REM sleep behavior disorder (RBD), a condition that is characterized by violent movements and nightmares during REM sleep [77]. In a small series of 13 MSA-C patients, RBD was found to precede waking motor symptoms in 3 patients and was present at diagnosis in about 50% of cases [78]. Curiously, RBDs commonly improve along with disease progression [75]. Excessive daytime sleepiness has been reported in about 25% of MSA-C patients, and might stem from low quality of sleep and/or dysfunction in neuronal pathways of arousal [79,80].

Many authors described cognitive impairment, involving executive functions and verbal learning in MSA-C [81-86]*.* These deficits are probably due to frontal atrophy [82,84] and white matter networks disruption [81,87].

Finally, psychiatric syndromes such as depression [86]*,* anxiety [85] and pathological laughing and crying [88], affect MSA-C patients more frequently than the general population.

### Diagnosis

According to current consensus diagnostic criteria [27], three different categories of increasing diagnostic certainty were defined. A definite diagnosis of MSA requires post-mortem examination. Probable MSA-C is defined by the presence of cerebellar ataxia and either limb ataxia, cerebellar dysarthria or cerebellar oculomotor dysfunction, together with autonomic dysfunction in the form of urinary incontinence and erectile dysfunction or severe orthostatic hypotension defined as a drop in systolic or diastolic blood pressure of 30 mmHg and 15 mmHg within 3 minutes after standing, respectively.

If only a mild autonomic dysfunction is found, possible MSA-C can be diagnosed taking into account pyramidal signs, parkinsonism, and imaging findings. T2-weighted MRI can reveal the so called “hot cross bun sign”, a cruciform hyperintensity in the ventral part of pons. This is one of the most distinctive although not pathognomonic imaging findings in MSA-C and has been reported in up to 81.4% of patients [64]. It is due to demyelation and fibrosis of transverse fibers in pons and it can be assessed also in other pathologies involving the pons, such as different types of SCAs, particularly SCA 2 [89]. Middle cerebellar peduncle hypointensity, lateral putaminal rim hyperintensity and putaminal hypointensity, are also considered in the diagnosis of possible MSA-C.

Diffusion weighted imaging (DWI) and diffusion tensor imaging (DTI) are MRI techniques that are considered still investigational in the latest MSA guidelines [27]. They can detect early alterations in the infratentorial region of MSA patients [90,91] and show a higher sensitivity compared to T2-weighted MRI for the “hot cross bun sign” [92]. Because of its high sensitivity, DTI might be helpful for the differential diagnosis in patients with cerebellar symptoms even in the earlier stages of MSA-C, (see below). DWI and DTI have also been proposed as possible markers for the progression of the disease [93].

Striatal dopaminergic denervation or glucose hypometabolism assessed with SPECT or PET, respectively, in the presence of cerebellar symptoms suggests the diagnosis of MSA-C rather than other forms of late onset ataxia (Table 1).

**Table 1:** **Diagnosis of MSA-C, modified from Gilman et al., 2008 [** 27 **]**

**Probable MSA-C:**	**Possible MSA-C:**
A sporadic progressive, adult (>30y)- onset disease characterized by:	A sporadic progressive, adult (>30y)- onset disease characterized by:
• Autonomic failure involving urinary incontinence (inability to control the release of the urine from the bladder, with erectile dysfunction in males) or an orthostatic decrease of blood pressure with 3 min of standing by at least 30 mmHg systolic or 15 mmHg diastolic	• Cerebellar syndrome
	• At least one feature suggesting autonomic dysfunction (otherwise unexplained urinary urgency, frequency, incomplete bladder emptying, erectile dysfunction, in males, or significant orthostatic blood pressure decline that does not meet the level required in probable MSA-C)
• Cerebellar syndrome	• At least one of the following feature:
	○ Babinski sing with hyperreflexia
	○ Stridor
	○ Parkinsonism (bradykinesia and rigidity)
	○ Atrophy on MRI of putamen, middle cerebellar peduncle, or pons
	○ Hypomethabolism on FDG-PET in putamen
	○ Presynaptic nigrostriatal dopaminergic denervation on PET or SPECT

Warning symptoms, also called “red flags”, may facilitate the diagnosis of MSA (Table 2) [94]. Disease progression can be quantified using the Unified MSA Rating Scale (UMSARS) [95]. It involves assessment of the activities of daily living (ADL) (UMSARS I), the motor function (UMSARS II), a simple standing test to determine the presence and magnitude of OH (UMSARS III) and a global disability scale (UMSARS IV)

**Table 2:** **Red flags for MSA, reproduced with the courtesy of Wiley and Sons** [85]

**Red flag**	**Definition**
Early instability with recurrent falls	within 3 years of disease onset
Rapid progression	“wheelchair sign”: dependent < 10 years from disease onset
Orofacial dystonia	based on clinical judgment
Camptocormia	prolonged episodes of forward trunk flexion
Pisa syndrome	prolonged episodes of lateral trunk flexion
Disproportionate antecollis	severe neck flexion, minor flexion elsewhere
Contractures of hands or feet	excluding Dupuytren’s or contracture due to other known cause
Jerky tremor	irregular postural or action tremor of the hands and/or fingers with definite myoclonus
Diurnal inspiratory stridor	based on clinical judgment
Nocturnal inspiratory stridor	based on clinical judgment
Inspiratory sighs	involuntary deep inspiratory sighs/gasps
Severe dysphonia	based on clinical judgment
Severe dysarthria	based on clinical judgment
Severe dysphagia	based on clinical judgment
REM sleep behavior disorder	intermittent loss of muscle atonia and appearance of elaborate motor activity (striking out with arms in sleep often with talking/shouting) associated with dream mentation
Sleep apnoea	prolonged arrests of breathing
Excessive snoring	increase from premorbid level, or newly arising
Cold hands/feet	new development of coldness and color change – purple/blue – of extremities, with blanching on pressure and poor circulatory return
Raynaud’s phenomenon	new emergence of painful “white fingers”
Emotional incontinence – crying	Inappropriate crying without sadness
Emotional incontinence – laughing	Inappropriate laughing without mirth
Past history of documented hypertension	based on clinical judgment

.

### Differential diagnosis

MSA-like cerebellar symptoms, are shared by many other primary and secondary cerebellar disorders. Secondary ataxia can be caused by toxins, infections, tumors, vitamin deficiency and several other pathologies. Primary diseases can be caused by mutations (spinocerebellar ataxias (SCAs) and Fragile-X related tremor and ataxia syndrome (FXTAS)), or remain idiopathic.

Alcohol is a common cause of ataxia, with the percentage of alcoholics developing ataxia ranging from 11% to 27% [96,97]. The possible relation between alcohol intake and cerebellar symptoms should be carefully assessed for a correct diagnosis. An improvement of symptoms after alcohol withdrawal is strongly indicative for alcoholic ataxia [98,99].

Several drugs can cause cerebellar damage [98,100-103]. It is, therefore, important to evaluate all medications taken by the patients, keeping in mind their possible adverse effects.

An important cause of late onset ataxia is a paraneoplastic syndrome. Small cell lung cancer, ovarian cancer, breast cancer, and Hodgkin's lymphoma are most usually associated with cerebellar injury [98]. This damage is commonly related with the production of circulating antineural antibodies directed against antigens expressed by neoplastic cells [104,105]. Peripheral blood testing, combined with the assessment of rapid progressive ataxia and systemic symptoms such as fever, malaise and anorexia in the absence of cerebellar abnormalities at MRI at the onset of motor symptoms, usually leads to the diagnosis of paraneoplastic cerebellar ataxia within 6 months [104]. Vitamin deficiency, superficial siderosis and infections can also cause ataxia (reviewed by *Klockgether, 2010*) [98].

Finally, ataxia can also be associated with autoimmune disorders. Anti-glutamate decarboxylase (GAD) antibodies positive ataxia is more frequent in diabetic patients [106] and in patients with polyglandular autoimmune disorder [107]. Gluten ataxia is a sporadic form of ataxia developing after chronic ingestion of gluten, with or without association with coeliac disease. Gluten ataxia is diagnosed if antigliadin antibodies are found in ataxic patients in the absence of other potential causes [108,109]. Anti-transglutamminase (TG) autoantibodies can also be found in patients with gluten ataxia. In particular, antibodies directed against TG 6, the most abundant TG isoform in the CNS, are specifically associated with gluten ataxia [110].

The collection of an accurate family history is important, but not always sufficient, to rule out genetic forms of ataxia. Late onset autosomal dominant SCAs, such as SCA6 [111,112], may be difficult to diagnose if the affected relatives died before developing the illness. In a study, genetic analysis was performed in patients with negative family history who developed ataxia when they were at least 25 years old. SCA6 was the predominant genetic form, but also mutations associated with SCA2 and SCA3 were found in few patients [113]. It is worth noting that in Asiatics and Africans SCA2 and SCA3 mutations cause L-DOPA-responsive parkinsonism [114,115].

A premutation of the *FMR1* gene causes a late onset form of ataxia called FXTAS. This disease is characterized by cerebellar ataxia, postural and intentional tremor, dementia, neuropathy, and several psychiatric manifestations [116]. A large investigation by Kamm and coworkers (2005) [117] showed that FMR1 premutation-associated ataxia is distinct from MSA-C. Nevertheless, the possible diagnosis of FXTAS has to be considered especially in the presence of slow disease progression, neuropathy and dementia. These clinical features are common among patients with FXTAS, and their assessment may help to differentiate the two disorders.

Most of the above conditions can be diagnosed with an accurate history. It is more difficult to differentiate MSA-C with sporadic adult onset ataxia (SAOA). SAOA, also known as idiopathic late onset cerebellar ataxia (ILOCA), is a rare form of ataxia, with a prevalence of about 8.4 *per* 100000 [118]. The age at onset for both MSA-C and SAOA is around 50 years [118]. However, the mean survival time is much longer in SAOA than in MSA-C. SAOA is not only associated with a better prognosis but also with a delayed deterioration in ADL. Patients with MSA require a wheelchair within 5 years of onset [64]; in contrast, about half of patients with SAOA can still walk unaided after 12 years [113,119].

A reliable differential diagnosis between SAOA and MSA-C can usually be performed years after the onset. Within 5 years from the diagnosis, about ¼ of patients with idiopathic late onset cerebellar ataxia are diagnosed as affected by MSA-C [113,120,121]. This can be easily explained considering that the presence of autonomic and motor signs is mandatory for the diagnosis of MSA-C [27] and that autonomic dysfunction becomes manifest only 2–2.5 years after the first cerebellar signs [64,70]. Initial clinical presentation do not help much in the differential diagnosis. Although nystagmus, gaze paralysis, decreased or absent ankle reflex are more common in SOAO [121,122], many motor and non-motor symptoms [123], RBD [75,122] and erectile dysfunction [68,122], are shared by MSA-C and SAOA.

The differential diagnosis between SAOA and MSA-C has been the subject of several studies. However, the large majority of these studies compared fully developed MSA and SAOA, whereas the differential diagnosis is particularly difficult in the early phase of the disease. T2-weighted MRI is used for the diagnosis of MSA [27] and has good specificity in differentiating fully developed MSA-C from SAOA [124]. Other MRI based techniques were used for the differential diagnosis between MSA-C and other ataxias, with interesting results. Proton MR spectroscopy imaging distinguished MSA-C from SCA2 on the basis of different levels of lactate in cerebellum [125]. DTI yielded a different fractional anisotropy in MSA-C and many other types of ataxia, but not SAOA [126]. A more valuable diagnostic tool is the measurement of cerebral blood flow in the pons by Fine-STR, which is lower in MSA-C than in SAOA [127].

In MSA-C there is loss of motor units innervating the external anal and urethral sphincter muscles reflecting degeneration of Onuf’s nucleus. These changes together with detrusor hyperreflexia account for early neurogenic bladder incontinence in MSA-C and they may help differentiate MSA-C from other sporadic late onset cerebellar ataxias in the first 5 years after the onset of the disease [128]. Sphincter denervation can be evaluated by means of anal sphincter electromyography (EMG) and urethral sphincter EMG [128-132]. The former is better tolerated by patients [129]. However, anal sphincter EMG is not highly sensitive in the early course of the disease, and it was positive only in 52% of patients with disease duration shorter than 1 year [130].

Measurements of proteins and monoamine metabolites in the cerebrospinal fluid (CSF) are particularly valuable in the diagnosis of chronic neurodegenerative disorders. Putative CSF biomarkers include neurofilament light chain (NFL), phosphorylated neurofilament heavy chain, α-synuclein, β-amyloid, tau, the noradrenaline metabolite, 3-methoxy-4-hydroxyphenylethylenglycol (MHPG), and the dopamine metabolites, dihydroxyphenylacetic and homovanillic acids [58,133-136]. Abdo et al. (2006) [137] have found that a combined measurement of NFL, MHPG and tau has 100% specificity in differentiating between MSA and SAOA. Interestingly, the CSF obtained from MSA patients promotes aggregation of α-synuclein to a greater extent than the CSF obtained from SCAs patients [138].

For the diagnosis of MSA-C, EMG and CSF analysis are generally not recommended [27], because they are not helpful in full blown cases and have not been jet validated in early cases.

### Treatment

Currently, the only treatments available for MSA-C are symptomatic. There are no approved drugs that can influence the disease course. The management of all possible manifestations of MSA is described in detail elsewhere [139-141]. Here, we will offer a brief description of the main therapeutic options that are currently available and will focus on new drugs under development.

#### Symptomatic treatment

Parkinsonism associated with MSA-C is treated with L-DOPA. Only half of patients with MSA-C respond to L-DOPA and dyskinesias, usually facial, are a common side effect [65]. When starting L-DOPA, a responsiveness test should be made with escalating doses in the first 3 months (up to a daily dose of 1000 mg if needed and tolerated) [142].

Currently, there is no treatment for cerebellar ataxia. The hypothesis of a possible effect of anticholinergic drugs is supported by the evidence that nicotine, which stimulates nicotinic cholinergic receptors in the CNS, can cause a reversible worsening of ataxic symptoms [10,143].

Urinary problems could be managed with drugs, at least at the beginning of the disease. The antimuscarinic drug, oxybutinine, reduces detrusor hyperreflexia and sphincter-detrusor dyssynergia, with positive effects on urgency and frequency [128,144]. Urinary retention is treated with α1-adrenergic antagonists, such as prazosin and moxysylate. All these drugs have important adverse effects that could limit their use. If postvoid residual volume exceeds 150 ml, although potentially difficult for ataxic patients, clean intermittent self-catheterization is indicated [144]. Many patients will eventually require a suprapubic catheter.

Orthostatic hypotension can be managed with non-pharmacological strategies. Adequate hydration of the patient has to be guaranteed [145]. Moreover, the patients should avoid large meals and increase sodium intake. Sitting or lying if feeling dizzy is recommended; if this is not possible, crossing legs, contracting thighs, and bending over can be helpful [146]. Abdominal and leg elastic garments are also effective in reducing orthostatic hypotension. Head-up tilt while sleeping reduces cerebral hypertension and increases circulatory volume within 1 week [140].

If these non-pharmacological measures fail, medical treatment is needed. Fludrocortisone is a potent agonist of mineralcorticoid receptors and increases blood pressure by enhancing sodium reuptake in the kidney. Alternatively, patients may be treated with the α1 adrenergic agonist, midodrine, or with the recently FDA approved adrenergic prodrug, droxidopa [147]. Finally, the vasopressin analogue, desmopressin, reduces nocturnal polyuria and morning hypotension [148].

Patients with postprandial hypotension may gain benefit by treatment with somatostatin analogues [141], which likely act by inhibiting the release of vasoactive gastrointestinal peptides [149].

Constipation is managed with high-fiber diet and, if necessary, macrogol-water solution.

Physiotherapy, speech therapy and occupational therapy may help patients coping with their disease [141].

#### New treatments

In the last years, many drugs have been tested as potential disease modifiers in MSA in general and MSA-C in particular. A growing body of evidence suggested efficacy in transgenic MSA mouse models [17]. However, growth hormone (GH) [150], the antiglutamatergic drug, riluzole [151], minocycline [152], rifampicin [153], and lithium [154] all failed to slow or halt disease progression in humans. A common feature of clinical trials with all these drugs, was to include patients in advanced stages, with fewer chances of gaining benefit by putative neuroprotective agents.

A Korean publication [155] reported beneficial effects of autologous bone marrow derived mesenchymal stem cells (MSC) in MSA patients, however these results have not been replicated so far and therefore remain experimentally until further confirmation studies are completed. Finally, some interesting results were obtained by intravenous infusion of immunoglobulins, which are known to exert anti-inflammatory activity [156]. Seven MSA patients have been treated for 6 months and showed an improvement in UMSARS I and UMSARS II subscales, whereas UMSARS III and UMSARS IV remained unchanged [157]. This study was carried out on a small number of patients, without a control group. However, bearing in mind that a large deterioration in UMSARS is usually seen in one year [158], results are promising. In addition, α-synuclein lowering strategies have shown efficacy in preclinical synucleinopathy models, thus raising the possibility that these strategies may ultimately arrest disease progression in MSA [159].

## Conclusions

Further advances in the diagnosis and treatment of MSA will go hand in hand. Novel neuroprotective strategies will be tested with the aid of novel diagnostic tools that allows an earlier start of the treatment.

Many ancillary exams with possible diagnostic value, such as MRI, anal sphincter EMG, CSF analysis and GH testing have been evaluated in MSA compared to other diseases. In our opinion, it becomes necessary to apply these diagnostic tools in patients with idiopathic cerebellar ataxia [113,120,121]. Hopefully, this new techniques will facilitate a differential diagnosis between MSA-C and SAOA at earlier time points. Clearly much is changed and many steps forward have been taken since Quinn portrayed MSA as a beast, in 1989 [160].

## Abbreviations

ADL: Activities of daily living
CSF: Cerebrospinal fluid
DARPP-32: Dopamine and c-AMP-regulated phosphoprotein-32
DWI: Diffusion weighted imaging
DTI: Diffusion tensor imaging
EMG: Electromyography
FMR1: Fragile-X mental retardation 1
FXTAS: Fragile X related tremor and ataxia syndrome
GCIs: Glial cytoplasmic inclusions
GH: Growth hormone
ILOCA: Idiopathic late onset cerebellar ataxia
L-DOPA: Levodopa
MHPG: 3-methoxy-4-hydroxyphenylethylenglycol
MSA: Multiple system atrophy
MSA-C: Cerebellar type MSA
MSA-P: Parkinsonian type MSA
MSC: Mesenchymal stem cells
NCIs: Neuronal cytoplasmic inclusions
NFL: Neurofilament light chain
NNIs: Neuronal nuclear inclusions
OPCA: Olivopontocerebellar atrophy
RBD: REM sleep behavior disorder
SAOA: Sporadic adult onset ataxia
SCAs: Spinocerebellar ataxias
SND: Striatonigral degeneration
SNPs: Single nucleotide polymorphisms
TG: Transglutaminase
TLRs: Toll-like receptors
UMSARS: Unified MSA Rating Scale
